# DNA Methylation Changes More Slowly Than Physiological States in Response to Weight Loss in Genetically Diverse Mouse Strains

**DOI:** 10.3389/fendo.2019.00882

**Published:** 2019-12-20

**Authors:** Chantle R. Edillor, Brian W. Parks, Margarete Mehrabian, Aldons J. Lusis, Matteo Pellegrini

**Affiliations:** ^1^Department of Human Genetics, University of California, Los Angeles, Los Angeles, CA, United States; ^2^Department of Nutritional Sciences, University of Wisconsin-Madison, Madison, WI, United States; ^3^Department of Medicine/Division of Cardiology and Department of Human Genetics, University of California, Los Angeles, Los Angeles, CA, United States; ^4^Department of Molecular, Cell, and Developmental Biology, University of California, Los Angeles, Los Angeles, CA, United States

**Keywords:** obesity, mouse strains, DNA methylation, weight loss, liver

## Abstract

Responses to a high fat, high sucrose (HFHS) diet vary greatly among inbred strains of mice. We sought to examine the epigenetic (DNA methylation) changes underlying these differences as well as variation in weight loss when switched to a low-fat chow diet. We surveyed DNA methylation from livers of 45 inbred mouse strains fed a HFHS diet for 8 weeks using reduced-representation bisulfite sequencing (RRBS). We observed a total of 1,045,665 CpGs of which 83 candidate sites were significantly associated with HFHS diet. Many of these CpGs correlated strongly with gene expression or clinical traits such as body fat percentage and plasma glucose. Five inbred strains were then studied in the context of weight loss to test for evidence of epigenetic “memory.” The mice were first fed a HFHS diet for 6 weeks followed by a low-fat chow diet for 4 weeks. Four of the five strains returned to initial levels of body fat while one strain, A/J, retained almost 50% of the fat gained. A total of 36 of the HFHS diet responsive CpGs exhibited evidence of persistent epigenetic modifications following weight normalization, including CpGs near the genes *Scd1* and *Cdk1*. Our study identifies DNA methylation changes in response to a HFHS diet challenge that revert more slowly than overall body fat percentage in weight loss and provides evidence for epigenetic mediated “memory.”

## Introduction

Epigenetic regulation, such as DNA methylation, was hypothesized and recently confirmed to play a role in the development of obesity in humans and rodents (1). The interactions between diet, genetic background, and epigenetic regulation are becoming better understood, with evidence suggesting that DNA methylation can be a causal factor in obesity (2). In rodents (where the effect of environment is more controlled), studies have identified specific DNA methylation changes in the liver at genes involved in metabolism (*Scd1, GK/glycerol kinase, L-PK/L-pyruvate kinase, Mttp*) as well as cell-cycle regulation (*Cdkn1a)* (3–6). Additional evidence suggests that human DNA methylation changed by as hepatic insulin resistance. Caloric restriction in humans and mice protects against aging associated pathologies like type 2 diabetes, heart disease, and hepatocellular carcinoma, while obesity increases this susceptibility (7–9). These changes are known to be mediated in part by DNA methylation, a biomarker reproducibly associated with chronological aging. These variable epigenetic loci are often used to fit tissue-specific biological clocks of “epigenetic aging” in association with biometrics like body mass index (BMI) (10). Obesity-associated DNA methylation changes have been also been observed to accelerate “epigenetic aging” in the liver and are not acutely reversible by interventions like bariatric surgery (10–12).

A clinically relevant aspect of such studies relates to the context of weight loss and whether the effects of dietary challenge and obesity remain. In human surveys of DNA methylation during weight loss, it was observed that modest but widespread changes to DNA methylation were found near known obesity and diabetes genes in liver and adipose (13). Other studies identified DNA methylation differences near known obesity genes predicting high or low responses to therapies like caloric restriction, exercise, or surgical interventions (14–21). It has been hypothesized that these methylation changes may be involved in a “memory” mechanism of weight-regain or persistent accelerated epigenetic aging after weight loss (22). Studying chromatin accessibility using FAIRE-seq in a mouse model of diet-induced obesity, Leung and colleagues observed strain specific evidence for “memory” using a weight loss study design (23). On the other hand, Siersbaek and colleagues found no evidence for epigenetic memory following 8 weeks of weight loss as assessed by H3K27Ac marks (24).

Here, we surveyed a large panel of genetically diverse inbred strains of mice previously shown to vary in response to a high-fat, high-sucrose (HFHS) diet using reduced-representation bisulfite sequencing (RRBS). We previously profiled the methylomes of over 100 strains on a chow diet to identify genetic variation influencing liver DNA methylation (25). To study the effect of diet on liver methylation patterns, we performed RRBS in a subset of strains after 8 weeks of obesity. The liver is a relevant tissue in obesity studies as it coordinates with adipose tissue to regulate glucose, insulin, and lipid levels. In human studies of epigenetic aging across various tissues, only the liver showed a significant correlation between accelerated epigenetic aging and BMI (10). Our analysis resulted in the identification of 83 CpGs corresponding to 62 loci exhibiting significant changes in methylation in a majority of strains in response to diet. We then asked how these sites respond to a reversion to a low fat chow diet and whether they maintain the “memory” of the HFHS diet state despite weight loss.

## Materials and Methods

### Animals and Experimental Design

A genetic study of diet-induced obesity was previously reported, where 100 diverse inbred mouse strains of both sexes were fed a high-fat (32.5% kcal from corn oil and butter), high-sucrose (25% kcal) diet for 8 weeks (26). We refer to this collection of strains as the Hybrid Mouse Diversity Panel (HMDP). A subset of 45 strains from this study were selected based on the availability of age-matched males of the same strain fed a chow-only (18% kcal from fats) diet for 8 weeks. All mice were obtained from the Jackson Laboratory and the animal protocol for the study was approved by the Institutional Care and Use Committee (IACUC) at University of California, Los Angeles. The HFHS diet was purchased from Research Diets (D12266B).

A subset of 5 HMDP strains (A/J, BALB/cJ, C3H/HeJ, C57BL/6J, and DBA/2J) was used as a validation and weight loss discovery cohort. These strains were selected in order to study the variability in weight loss phenotypes based on their body fat responses to HFHS diet. BALB/cJ was found to be a low responding strain in terms of body fat gain, A/J, C57BL/6J, and C3H/HeJ responding moderately, and DBA/2J animals with the greatest response to 8 weeks of HFHS diet (26). Animals from all 5 strains were fed either HFHS (*n* = 16/strain) or chow (*n* = 8/strain) diets for 6 weeks. Half of the HFHS group was then returned to chow for 4 weeks and treated as a weight loss phenotype (*n* = 8/strain), while the remaining animals remained on chow- or HFHS-only diets. In both the obesity and weight loss studies, body composition was measured bi-weekly throughout the course of the diet via NMR. At the time of sacrifice, metabolic phenotypes such as glucose tolerance, serum insulin, and lipids were measured. The log-value of these clinical measurements were used for correlation analyses with methylation changes.

### Liver Methylome Profiling and Differential Methylation Analysis

We isolated genomic DNA from 30 to 50 mg of whole liver from 16-week old animals fed chow or HFHS diet. For each of the 45 strains, DNA from 2 to 3 males per strain was pooled for library preparation, producing epigenetic profiles that represent the aggregate methylation profiles for a strain. Genomic DNA was isolated using the Qiagen AllPrep DNA/RNA Mini Kit. When anatomy of the frozen tissue was discernible, 30–50 mg samples were cut from the middle of the left caudate lobe. Tissues were homogenized in RLT Plus Buffer in a Qiagen TissueLyser at 50 Hz for 2 min. DNA concentrations were measured using the Qubit fluorometer with the DS DNA kit. RRBS libraries were prepared according to previously published protocols (8). We initially combined and sequenced the genomic DNA of 2–3 animals for the initial set of 45 HMDP libraries and sequenced 3 individual animals per diet in the weight loss cohort. One-hundred ng of genomic DNA was digested with the methylation-insensitive restriction enzyme MspI, and fragments of length from 50 to 400 bp were selected. We sequenced each library to an average depth of 20 million reads per sample on the Illumina HiSeq4000 platform. Sequencing reads were mapped to mouse genome build mm 10 using an RRBS specific aligner, BSSeeker2 (27). We observed 45% mappability across all samples. CpGs on opposite strands were not merged and maintained as separate sites. We filtered the cytosines based on CG context, coverage, and representation across all samples. This yielded >1 million CpGs with 10x coverage across at least 70% of samples. CG-SNPs, where one allele of a genetic variant is also a CpG site, were removed. CG-SNPs were identified by cross referencing profiled CpGs with HMDP SNPs imputed by Bennett et al. (28). For each remaining CpG site with 10x or greater coverage, the methylation level was calculated by computing the ratio of methylated to unmethylated reads at each site. This proportion represents the percentage of cells in the sampled tissue with a methylated cytosine. We removed CpGs with low variance (row variance <0.01) across all strains and diets. For each CpG site in the dataset we first calculated the intra-strain change in methylation between HFHS and chow diets. We compared the means at each site between diets using a two-tailed paired *T*-test. To account for multiple comparisons, we calculated a Benjamini-Hochberg corrected FDR-adjusted *p*-value. FDR-significant sites had a differential FDR <0.1 which corresponded to an unadjusted *p*-value of 5E-5. We permuted the diet labels and performed 1,000 permutations at every analyzed CpG.

### Genomic and Functional Annotation

Significant CpGs were annotated to nearby genes using GREAT (29). Annotated regions correspond to 1,000 kb surrounding a gene's TSS. Once annotated, GREAT performs pathway enrichment using GO and KEGG terms. A set of 100,000 randomly selected CpGs were selected from the data as background for enrichment.

### Statistical Analyses

All statistical analyses were performed using the Python 3.0 packages Pandas and NumPy for processing DataFrames, and Scipy Stats for statistical test functions like *t*-tests, FDR corrections, permutation, and calculating correlation coefficients values reported are Pearson's coefficient. Phenotypes are log-normalized.

## Results

### Diet-Responsive Methylation Changes in a Subset of the HMDP

**Figure 1:**
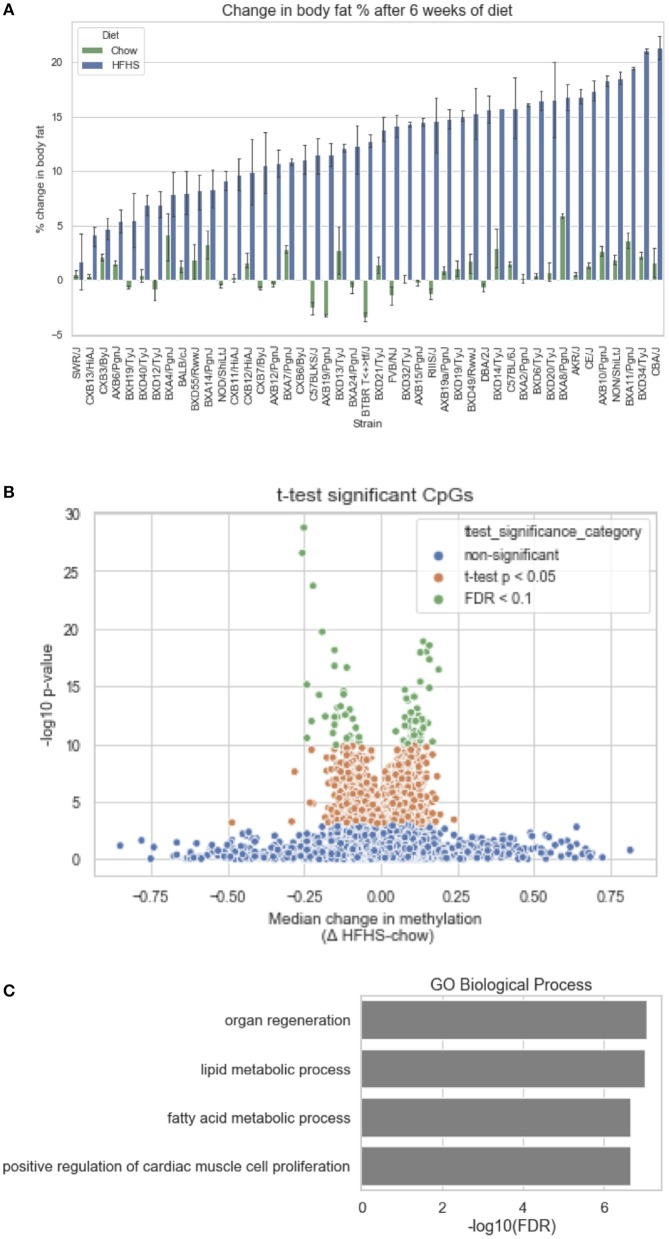
Differential methylation of CpGs in response to HFHS diet. Forty-five genetically diverse mouse strains were fed an obesifying high-fat, high-sucrose (HFHS) or chow diet for 8 weeks. **(A)** Shows the strain-specific body fat changes after 6 weeks of diet. Such strains such as DBA/2J and C57BL/6J are high responders to HFHS diet and increase adiposity 15% after 6 weeks compared to chow diet, while other stains like BALB/cJ gain much less. Data shown are mean ± SD (*n* = 2–3 mice/strain and diet). **(B)** Differential analysis identifies 83 significant (10% FDR) CpGs out of 161,742 variable CpGs. Out of 1,045,665 CpGs identified by RRBS, only a subset varied between samples. **(C)** Annotation of significant CpGs to nearby genets and GO Term Pathway enrichment, x axis values represent -log_10_(FDR) values of term enrichment.

We first identified CpGs that change methylation levels in response to a high-fat, high-sucrose (HFHS) diet. The obesogenic response is highly variable among the subset of strains selected, with some strains responding with low percentage of body fat gain and some high (Figure 1A). We performed reduced representation bisulfite sequencing (RRBS) on DNA isolated from liver and observed a total of 1,045,665 CpGs with 10x coverage across at least 70% of samples. A majority of CpGs measured did not vary between strains, as they tended to be ubiquitously unmethylated in all samples and were excluded from the analysis. We performed paired sample *t*-tests on the methylation levels at 161,742 variable CpGs between chow and HFHS groups composed of 45 HMDP strains (Figure 1B). This analysis identified 83 CpGs that significantly change in response to HFHS diet group with an FDR <10% (Table 1 and Supplementary Figure 1). There were 32 CpGs with decreased methylation levels in the HFHS group (−7 to −25.5%) and 51 CpGs that increased in methylation (+5 to +19%) in the HFHS group. We ran 1,000 permutations of the *t*-test by randomizing the diet label to identify the false positive rate. The average number of CpGs where the permutation *p*-value was less than the FDR cut-off, <5E-5, was 25.22 (95%CI = 24.81–25.64), suggesting that the inferred FDR rate may be closer to 30%.

**Table 1:** FDR significant CpGs changed by HFHS diet in 45 strains of mice.

**Chr**	**BP**	**Median change by HFHS**	***t*-test *p*-value**	**FDR**	**Annotated gene 1**	**Distance to TSS**	**Annotated gene 2**	**Distance to TSS**
1	73957398	0.14	6.28E-09	2.03E-04	Tns1	167049	Tnp1	−941500
1	89466797	0.1	1.46E-05	4.26E-02	Agap1	11987	D130058E05Rik	−463437
1	90962615	0.125	4.50E-05	9.11E-02	Rab17	7046	Prlh	9508
1	133264749	0.105	5.94E-06	2.28E-02	Plekha6	18653	Golt1a	−45074
1	151244113	−0.12	4.65E-07	4.18E-03	Ivns1abp	−100385	Hmcn1	−251063
10	63583522	0.05	1.53E-05	4.26E-02	Ctnna3	126013	Gm10118	343912
10	69248814	−0.1	3.79E-05	8.28E-02	Rhobtb1	35621	Cdk1	104124
10	69248832	−0.18	4.21E-06	2.03E-02	Rhobtb1	35639	Cdk1	104106
10	69248838	−0.15	4.53E-06	2.03E-02	Rhobtb1	35645	Cdk1	104100
10	69248844	−0.19	2.69E-09	1.09E-04	Rhobtb1	35651	Cdk1	104094
10	69248850	−0.2	6.51E-07	5.26E-03	Rhobtb1	35657	Cdk1	104088
10	69248853	−0.14	4.36E-06	2.03E-02	Rhobtb1	35660	Cdk1	104085
10	69248866	−0.15	8.52E-06	2.87E-02	Rhobtb1	35673	Cdk1	104072
11	51785947	0.115	4.89E-06	2.13E-02	Sar1b	22261	Phf15	71706
11	98877224	0.12	4.14E-05	8.82E-02	Wipf2	13587	Cdc6	−30927
11	106818615	0.105	1.44E-05	4.26E-02	Cep95	29341	Smurf2	101855
11	107265371	0.14	5.06E-06	2.13E-02	Nol11	−75991	Pitpnc1	205328
11	117233894	−0.15	4.34E-06	2.03E-02	43717	34234	Gm11733	−250474
11	117820356	0.11	7.08E-07	5.45E-03	Afmid	−5568	Syngr2	10689
11	117820370	0.15	1.51E-08	2.92E-04	Afmid	−5554	Syngr2	10703
11	120821045	−0.1	2.32E-06	1.34E-02	Fasn	3133	Dus1l	−24651
12	73838480	−0.225	6.33E-06	2.28E-02	Hif1a	−69424	Prkch	253684
12	73838512	−0.24	2.73E-05	6.61E-02	Hif1a	−69392	Prkch	253716
12	82399195	0.105	1.51E-05	4.26E-02	Sipa1l1	87847	Gm5435	97342
12	84769968	0.13	1.62E-08	2.92E-04	Npc2	3144	Isca2	−3302
12	85754916	0.08	4.16E-07	3.96E-03	Mfsd7c	8378	0610007P14Rik	69606
12	85975275	0.12	2.12E-06	1.27E-02	Tgfb3	103766	Ttll5	150566
13	46615383	0.1	2.96E-06	1.65E-02	C78339	−54139	Cap2	113536
14	63202824	0.155	7.48E-06	2.57E-02	Neil2	−9284	Gata4	42424
15	7187724	0.16	3.01E-08	4.86E-04	Lifr	33372	Egflam	210580
15	38302403	−0.14	1.94E-06	1.20E-02	Klf10	−1697		
15	38302475	−0.12	6.14E-07	5.23E-03	Klf10	−1769		
15	38302543	−0.07	3.57E-05	8.02E-02	Klf10	−1837		
15	57936054	−0.13	1.74E-06	1.13E-02	Wdr67	23856	Fam83a	−49365
15	85745965	0.08	4.49E-06	2.03E-02	Ppara	10190	Cdpf1	65731
15	85745974	0.09	1.05E-06	7.08E-03	Ppara	10199	Cdpf1	65722
15	85745987	0.13	2.04E-07	2.36E-03	Ppara	10212	Cdpf1	65709
16	24391817	0.09	4.27E-05	8.98E-02	Lpp	−1533		
16	34113282	0.1	2.51E-05	6.25E-02	Umps	−146245	Kalrn	400766
16	38432088	0.08	9.93E-06	3.15E-02	Pla1a	1057	Popdc2	69880
17	28065710	−0.07	2.51E-05	6.25E-02	Tcp11	14874	Anks1	156371
17	28065873	−0.15	5.21E-08	7.67E-04	Tcp11	14711	Anks1	156534
17	28065883	−0.09	5.14E-06	2.13E-02	Tcp11	14701	Anks1	156544
17	47442447	0.125	4.62E-05	9.23E-02	1700001C19Rik	−5073	Taf8	59840
19	44414923	−0.22	5.01E-11	2.70E-06	Scd1	−7215	Wnt8b	−78549
19	44414943	−0.25	3.18E-13	5.15E-08	Scd1	−7235	Wnt8b	−78529
19	44414944	−0.255	2.87E-12	2.32E-07	Scd1	−7236	Wnt8b	−78528
19	44414952	−0.15	1.33E-08	2.92E-04	Scd1	−7244	Wnt8b	−78520
19	44414953	−0.11	6.00E-08	8.09E-04	Scd1	−7245	Wnt8b	−78519
19	53610483	0.14	6.18E-06	2.28E-02	Smc3	10086	Rbm20	−66823
2	4891640	0.105	2.82E-05	6.72E-02	Sephs1	10077	Phyh	−27379
2	31488626	0.11	1.48E-05	4.26E-02	Ass1	18420	Fubp3	−84099
2	31511721	0.13	3.36E-05	7.73E-02	Ass1	41515	Fubp3	−61004
2	32417288	0.085	3.97E-05	8.56E-02	Ptges2	21393	Naif1	−33169
2	32417292	0.09	9.93E-06	3.15E-02	Ptges2	21397	Naif1	−33165
2	32928045	−0.24	2.66E-07	2.87E-03	Rpl12	−33514	Fam129b	51932
2	155185551	0.09	4.36E-05	9.03E-02	Dynlrb1	−50984	Itch	51993
2	160741022	0.075	3.39E-05	7.73E-02	Plcg1	9713	Zhx3	118609
2	174858446	−0.08	1.12E-05	3.47E-02	Edn3	97828	Gm14444	−151873
3	88568840	0.19	7.19E-08	8.94E-04	Ssr2	−10831	Ubqln4	15125
3	129421906	−0.115	3.68E-06	1.98E-02	Enpep	−89187	Elovl6	−110480
3	138224032	0.17	3.77E-05	8.28E-02	Adh7	6210	Adh1	−53462
4	8748959	0.13	2.21E-05	5.72E-02	Chd7	57595	Clvs1	−520358
4	41371302	0.125	3.96E-06	2.03E-02	Ubap1	22307	Kif24	93538
5	92365051	−0.1	2.23E-05	5.72E-02	Cxcl10	−16170	Nup54	70168
5	120483727	0.13	1.71E-05	4.68E-02	Sds	7197	Plbd2	19898
5	123029761	0.085	8.85E-07	6.23E-03	Orai1	14688	Morn3	17255
5	125147678	−0.11	3.21E-05	7.53E-02	Ncor2	31375	Fam101a	144232
5	125463847	0.1	4.73E-05	9.33E-02	Aacs	−11967	Bri3bp	22280
5	142593387	0.16	8.76E-09	2.36E-04	Mmd2	15413	Radil	−42290
5	142593399	0.08	9.27E-06	3.06E-02	Mmd2	15401	Radil	−42302
5	145871583	0.11	7.65E-07	5.62E-03	Cyp3a11	8381	Cyp3a44	−65710
5	146005723	0.16	3.51E-07	3.54E-03	Cyp3a25	3894	Cyp3a11	−125760
5	146005929	0.12	6.54E-06	2.30E-02	Cyp3a25	3688	Cyp3a11	−125966
5	149008580	−0.145	4.98E-05	9.70E-02	Hmgb1	44460	Gm15409	49303
7	80219975	0.1	6.03E-06	2.28E-02	Cib1	12838	Sema4b	33135
7	98352904	0.09	4.46E-05	9.11E-02	Tsku	8384	Acer3	−43367
8	119442446	0.14	1.14E-05	3.47E-02	Necab2	−4273		
8	122231359	0.135	5.38E-06	2.17E-02	Banp	280816	Gm22	−38210
9	21632184	−0.155	1.78E-05	4.80E-02	Smarca4	16016	Ldlr	−91392
9	42461997	0.115	6.25E-06	2.28E-02	Tbcel	10261	Tecta	−62069
9	46243778	−0.11	2.74E-05	6.61E-02	Apoa4	3083	Apoa5	−24855
9	108378240	0.11	2.13E-05	5.66E-02	1700102P08Rik	−14594	Usp4	30410

DNA methylation regulates gene expression at the promoters of genes but also at more distal enhancers, so we used the annotation tool GREAT, which assigns non-coding regulatory regions to nearby genes within 1 Mbp. This annotation identified 118 genes associated with the 83 HFHS diet-responsive CpGs. GO Term analysis of the annotated genes revealed significant enrichment for genes involved lipid metabolism and cellular replication (Figure 1C). Among lipid metabolism genes that were near the differentially methylated CpGs were *Ldlr, Elovl6, Fasn, Ppara*, and *Scd1*. Several of the 83 CpGs are proximal to the same genes, including *Cdk1*, cyclin dependent kinase 1, a mitosis checkpoint gene, and *Scd1*, a fatty acid biosynthesis gene.

**Figure 2:**
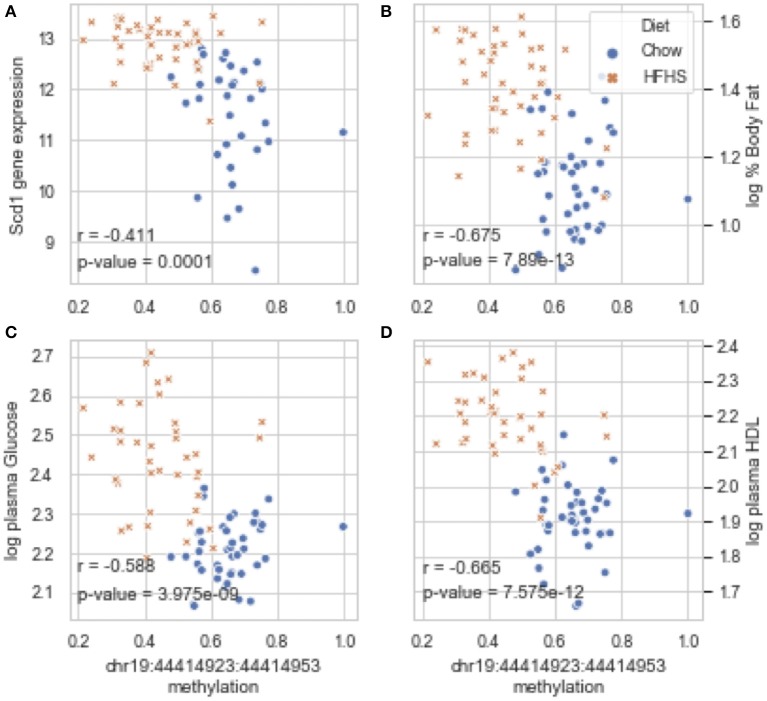
Diet-induced changes in methylation correlate with cis-regulated gene expression and clinical traits. **(A–D)** Show correlation plots between the average methylation levels of 5 correlated CpGs on chr19 and microarray transcript levels, body fat measured by NMR, and plasma glucose and HDL in 37 strains from the obesity HMDP. The blue dots represent the average methylation and trait measurements of a strain fed chow diet, and the orange crosses show data from the same strains fed HFHS diet.

Global liver gene expression from the obesity HMDP was previously quantified using Affymetrix HT_MG430A arrays (26). Expression values represent the average normalized probe intensity of 1-2 mice per strain, but not necessarily from the same animals pooled for RRBS. Thirty seven of the 45 HMDP strains had previously measured liver expression on both diets. Of the genes present on the array that were annotated to one of the 83 diet-responsive CpGs, 83 CpG methylation-transcript level correlations were significant with a *p*-value <0.05 (Table 2). This suggests that the diet-responsive methylation changes are likely associated with changes in nearby gene expression. Sixteen CpGs out of the 83 significant CpG-transcript correlations fell within <10 kbp of the gene's TSS and 53 of 85 CpGs were located within 50 kbp. In addition to gene expression, many CpGs correlate with clinical traits. Average methylation of the 5 CpGs annotated to *Scd1* inversely correlated with *Scd1* expression, body fat percentage, plasma glucose, and plasma HDL levels (Figure 2).

**Table 2:** Correlation of diet responsive CpG methylation levels with expression of nearby genes.

**CpG**	**Gene 1**	**R**	***p*-value**	**CpG**	**Gene 2**	**R**	***p*-value**
**METHYLATION-EXPRESSION CORRELATIONS WITH ANNOTATED GENES**							
chr1:73957398	Tns1	0.06	6.05E-01	chr1:73957398	Tnp1	0.14	2.51E-01
chr1:89466797	Agap1	0.31	9.50E-03				
chr1:90962615	Rab17	0.15	2.04E-01				
chr1:133264749	Golt1a	0.24	4.29E-02	chr1:133264749	Plekha6	0.42	2.47E-04
chr1:151244113	Ivns1abp	−0.34	5.72E−03				
chr10:69248814	Cdk1	−0.37	1.74E-03				
chr10:69248832	Cdk1	−0.46	8.75E-05				
chr10:69248838	Cdk1	−0.42	3.49E-04				
chr10:69248844	Cdk1	−0.55	1.14E-06				
chr10:69248850	Cdk1	−0.48	2.84E-05				
chr10:69248853	Cdk1	−0.42	3.06E-04				
chr10:69248866	Cdk1	−0.43	2.61E-04				
chr11:51785947	Sar1b	0.41	6.51E-04				
chr11:98877224	Cdc6	0.32	7.62E-03				
chr11:107265371	Nol11	0.16	1.74E-01	chr11:107265371	Pitpnc1	0.42	3.00E-04
chr11:117820356	Syngr2	−0.02	8.64E-01				
chr11:120821045	Dus1l	−0.34	5.45E-03	chr11:120821045	Fasn	−0.34	5.81E-03
chr11:117820370	Syngr2	−0.03	8.24E-01				
chr12:73838480	Hif1a	−0.04	7.63E-01	chr12:73838480	Prkch	−0.40	5.06E-03
chr12:73838512	Hif1a	0.02	8.86E-01	chr12:73838512	Prkch	−0.27	6.34E-02
chr12:84769968	Isca2	0.25	4.29E-02	chr12:84769968	Npc2	0.20	9.68E-02
chr12:85754916	Mfsd7c	−0.16	1.89E-01	chr12:85754916	0610007P14Rik	0.39	1.03E-03
chr12:85975275	Tgfb3	0.50	6.21E-06				
chr13:46615383	Cap2	0.45	1.39E-04				
chr14:63202824	Gata4	0.15	2.33E-01				
chr15:7187724	Lifr	0.53	1.55E-06				
chr15:38302403	Klf10	−0.41	4.35E-04				
chr15:38302475	Klf10	−0.33	4.42E-03				
chr15:38302543	Klf10	−0.28	1.76E-02				
chr15:85745965	Ppara	0.50	1.18E-05	chr15:85745965	Cdpf1	0.26	3.22E-02
chr15:85745974	Ppara	0.57	3.08E-07	chr15:85745974	Cdpf1	0.36	2.66E-03
chr15:85745987	Ppara	0.56	7.43E-07	chr15:85745987	Cdpf1	0.35	2.78E-03
chr16:24391817	Lpp	0.45	1.02E-04				
chr16:34113282	Umps	0.37	1.42E-03	chr16:34113282	Kalrn	0.03	8.30E-01
chr16:38432088	Pla1a	−0.41	3.47E-04	chr16:38432088	Popdc2	0.31	9.47E-03
chr17:28065710	Tcp11	−0.35	2.96E-03	chr17:28065710	Anks1	−0.38	1.20E-03
chr17:28065873	Tcp11	−0.38	1.20E-03	chr17:28065873	Anks1	−0.48	2.15E-05
chr17:28065883	Tcp11	−0.31	8.45E-03	chr17:28065883	Anks1	−0.41	4.28E-04
chr17:47442447	1700001C19Rik	0.23	6.07E-02	chr17:47442447	Taf8	0.43	2.73E-04
chr19:44414923	Wnt8b	−0.41	4.90E-04	chr19:44414923	Scd1	−0.59	1.13E-07
chr19:44414943	Wnt8b	−0.51	8.16E-06	chr19:44414943	Scd1	−0.54	2.09E-06
chr19:44414944	Wnt8b	−0.49	1.82E-05	chr19:44414944	Scd1	−0.54	1.76E-06
chr19:44414952	Wnt8b	−0.46	7.50E-05	chr19:44414952	Scd1	−0.49	2.28E-05
chr19:44414953	Wnt8b	−0.36	2.29E-03	chr19:44414953	Scd1	−0.49	1.64E-05
chr19:53610483	Smc3	0.40	6.23E-04				
chr2:4891640	Phyh	0.40	6.42E-04	chr2:4891640	Sephs1	0.37	1.71E-03
chr2:32417288	Ptges2	0.36	2.33E-03				
chr2:32417292	Ptges2	0.39	8.89E-04				
chr2:32928045	Fam129b	−0.48	5.13E-05				
chr2:155185551	Dynlrb1	0.45	7.10E-05	chr2:155185551	Itch	0.40	5.33E-04
chr2:174858446	Edn3	−0.11	3.63E-01				
chr3:88568840	Ssr2	0.62	7.50E-08	chr3:88568840	Ubqln4	−0.16	2.06E-01
chr3:129421906	Elovl6	−0.55	1.27E-06	chr3:129421906	Enpep	−0.50	1.34E-05
chr3:138224032	Adh1	0.20	1.13E-01	chr3:138224032	Adh7	0.20	1.12E-01
chr4:8748959	Clvs1	0.50	9.84E-05	chr4:8748959	Chd7	0.56	6.31E-06
chr4:41371302	Ubap1	0.36	2.25E-03				
chr5:92365051	Cxcl10	−0.20	8.92E-02	chr5:92365051	Nup54	−0.38	1.08E-03
chr5:120483727	Sds	−0.22	9.46E-02	chr5:120483727	Plbd2	−0.33	8.74E-03
chr5:123029761	Orai1	0.03	8.37E-01	chr5:123029761	Morn3	0.40	8.75E-04
chr5:125147678	Ncor2	−0.01	9.27E-01				
chr5:125463847	Aacs	0.29	1.68E-02				
chr5:142593387	Mmd2	0.17	1.55E-01				
chr5:142593399	Mmd2	0.17	1.68E-01				
chr5:145871583	Cyp3a44	0.48	3.12E-05	chr5:145871583	Cyp3a11	0.05	7.12E-01
chr5:146005723	Cyp3a11	−0.16	1.78E-01	chr5:146005723	Cyp3a25	0.19	1.20E-01
chr5:146005929	Cyp3a11	−0.13	3.08E-01	chr5:146005929	Cyp3a25	0.28	2.64E-02
chr5:149008580	Hmgb1	−0.37	1.99E-03				
chr7:80219975	Cib1	0.11	3.68E-01	chr7:80219975	Sema4b	0.39	9.49E-04
chr7:98352904	Tsku	−0.22	7.75E-02				
chr8:119442446	Necab2	0.42	4.30E-04				
chr8:122231359	Banp	−0.13	3.00E-01				
chr9:21632184	Ldlr	0.03	7.98E-01	chr9:21632184	Smarca4	−0.45	1.11E-04
chr9:42461997	Tecta	0.02	8.73E-01	chr9:42461997	Tbcel	0.42	2.67E-04
chr9:46243778	Apoa5	−0.25	4.03E-02	chr9:46243778	Apoa4	−0.07	5.65E-01
chr9:108378240	1700102P08Rik	0.33	5.03E-03	chr9:108378240	Usp4	0.36	2.44E-03

### Diet-Responsive Methylation Changes in a 5 Strain Model of Weight Loss

These 83 diet-responsive CpGs were further examined in a 5-strain study of diet-induced obesity followed by weight loss. The inbred strains were selected based on the variable responses to HFHS identified by Parks et al. (26). We fed mice HFHS for 6 weeks, then placed a subset back to chow for a diet-induced weight loss arm for an additional 4 weeks. At the end of 10 weeks, animals were sacrificed, and tissues collected for methylation profiling.

**Figure 3:**
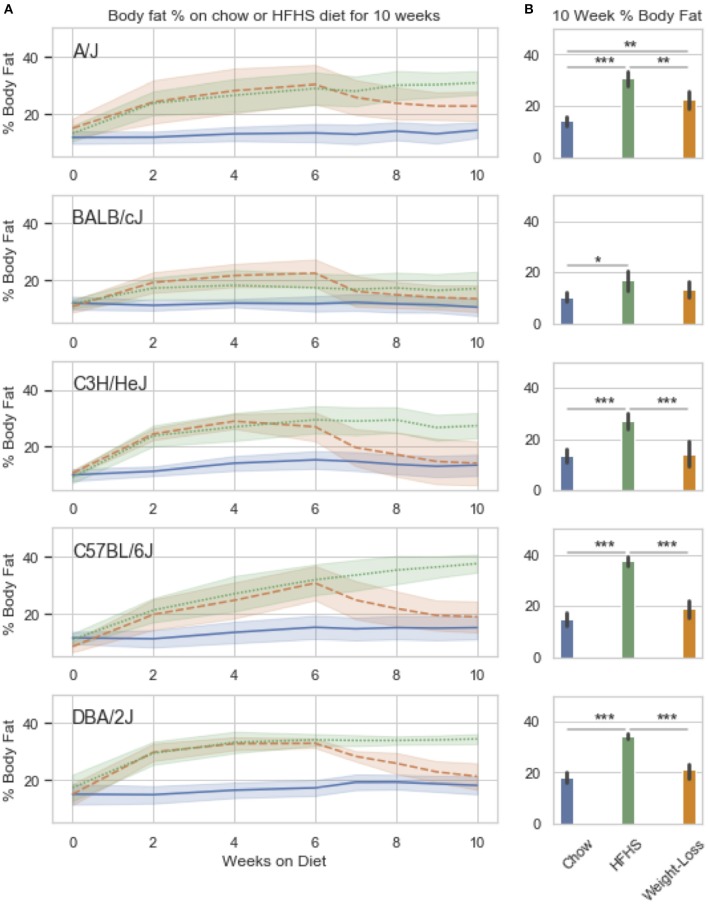
Weight loss and replication study. Five classical inbred strains (A/J, BALB/cJ, C3H/HeJ, C57BL/6J, and DBA/2J) were chosen based on a variable response to HFHS diet in previous studies. Twenty four 8 week old male mice were placed on HFHS (*n* = 16) or chow (*n* = 8) for 6 weeks, after which, a subset of 8 mice were placed back to chow (weight loss) for 4 weeks. Body fat percentage measured by NMR is shown over the course of 10 weeks in **(A)**. Data is mean ± SD. *T*-test comparisons of body weight and total adiposity at the end of the 10 week study show that only the A/J strain has not resolved diet-induced obesity after 4 weeks of diet **(B)**.

We observed strain specific changes in adiposity after 6 weeks of HFHS feeding, with BALB/cJ gaining the least and DBA/2J the most (Figure 3A). After reversion to weight loss diet for 4 weeks, four strains, A/J, C3H/HeJ, C57BL/6J, and DBA/2J, reverted to their chow adiposities and no longer had significantly more body fat than animals fed chow diet-only for 10 weeks (Figure 3B). When we examined DNA methylation changes by HFHS diet exposure, we confirmed that the diet-induced changes were robust across experiments. Three strains, BALB/cJ, C57BL/6J and DBA/2J, were present in both studies. We calculated the average change in methylation within each of the strains after HFHS diet in both studies then correlated the effects. The direction and magnitude of change in DNA methylation at HFHS-variable sites was highly reproducible between experiments (Supplementary Figure 2A). When correlated with body fat in each diet group, methylation specifically at the *Scd1* locus correlated the least with body fat in weight loss compared to chow or HFHS-only (Supplementary Figure 2B).

**Figure 4:**
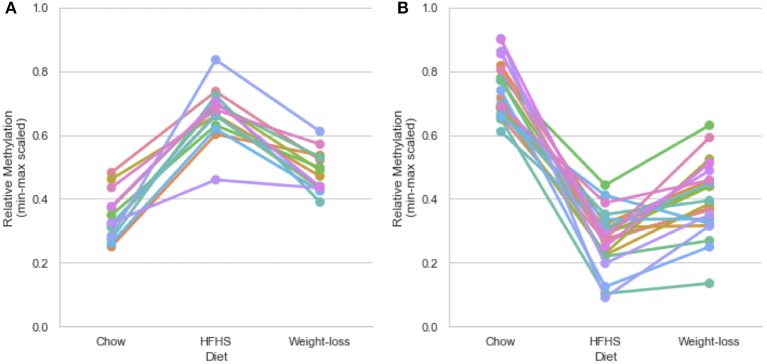
Persistently differentially methylated CpGs after weight loss. Thirty-six of 81 diet-responsive CpGs maintained a significant difference in methylation following 6 weeks of weight loss. Colored lines represent the average scaled methylation values of all 5 strains at singular diet-responsive CpGs (*n* = 15). Y-axis values are min-max scaled, which represents the absolute methylation percentage determined by RRBS relatively scaled to the minimum and maximum measurements of the same CpG across all strains and diets. (Measured methylation-minimum)/(maximum – minimum). **(A)** Shows examples of 14 persistently hypermethylated CpGs, and **(B)** shows examples of 22 hypomethylated CpGs.

We performed a two-way ANOVA on methylation levels at 81 of the diet-responsive CpGs between diet groups and strain variables. We identified 44 CpGs with a *p*-value <0.05 for the diet term. When we performed differential analysis by *t*-test in the weight loss group compared to chow-only animals, many of the diet-induced changes were non-significant, but a fraction of the sites remained differentially methylated (*p* < 0.05) (Figure 4). Thirty-six of 81 diet-induced CpGs examined in the second cohort did not revert to chow-only levels of methylation across all strains. Although significantly differentially methylated after 6 weeks, it should be noted that most of methylation differences in the weight loss group began to revert to chow-only levels. There were 14 persistently hypermethylated and 22 hypomethylated CpGs. All persistent hypomethylated CpGs were also significant by ANOVA (*p* < 0.05) across all 3 diet groups, and 11 of 14 persistently hypermethylated CpGs were significant. The genes associated with weight loss-persistent CpGs are listed in Table 3. CpGs associated with *Scd1* and *Cdk1* remain significantly hypomethylated following 4 weeks of weight loss.

**Table 3:** Diet responsive CpGs with incomplete methylation reversal following 4 weeks of weight loss.

**CpG**	**Gene 1**	**Gene 2**	**Median Chow methylation**	**Median HFHS methylation**	**Median Weight loss methylation**
**PERSISTENTLY HYPERMETHYLATED SITES**					
chr1:73957398	Tns1	Tnp1	0.75	0.84	0.73
chr11:106818615	Cep95	Smurf2	0.33	0.42	0.48
chr11:107265371	Nol11	Pitpnc1	0.40	0.52	0.44
chr12:85754916	Mfsd7c	0610007P14Rik	0.66	0.72	0.69
chr15:7187724	Lifr	Egflam	0.44	0.53	0.44
chr16:34113282	Umps	Kalrn	0.75	0.83	0.78
chr2:31488626	Fubp3	Ass1	0.46	0.62	0.53
chr2:31511721	Fubp3	Ass1	0.64	0.75	0.65
chr2:160741022	Plcg1	Zhx3	0.30	0.47	0.30
chr3:138224032	Adh1	Adh7	0.48	0.62	0.52
chr4:41371302	Ubap1	Kif24	0.65	0.77	0.74
chr5:123029761	Orai1	Morn3	0.18	0.23	0.20
chr5:125463847	Aacs	Bri3bp	0.46	0.50	0.40
chr5:145871583	Cyp3a44	Cyp3a11	0.74	0.86	0.79
**PERSISTENTLY HYPOMETHYLATED SITES**					
chr10:69248814	Rhobtb1	Cdk1	0.55	0.47	0.47
chr10:69248832	Rhobtb1	Cdk1	0.68	0.61	0.59
chr10:69248838	Rhobtb1	Cdk1	0.53	0.52	0.46
chr10:69248844	Rhobtb1	Cdk1	0.67	0.58	0.55
chr10:69248850	Rhobtb1	Cdk1	0.61	0.44	0.43
chr10:69248853	Rhobtb1	Cdk1	0.54	0.44	0.42
chr10:69248866	Rhobtb1	Cdk1	0.79	0.71	0.67
chr11:117233894	Gm11733	43717	0.93	0.89	0.89
chr11:120821045	Dus1l	Fasn	0.76	0.76	0.73
chr15:38302403	Klf10		0.22	0.20	0.18
chr15:38302475	Klf10		0.24	0.19	0.16
chr15:38302543	Klf10		0.15	0.15	0.14
chr17:28065710	Tcp11	Anks1	0.31	0.25	0.25
chr17:28065873	Tcp11	Anks1	0.44	0.43	0.44
chr17:28065883	Tcp11	Anks1	0.40	0.40	0.33
chr19:44414923	Wnt8b	Scd1	0.54	0.46	0.42
chr19:44414943	Wnt8b	Scd1	0.81	0.77	0.66
chr19:44414944	Wnt8b	Scd1	0.83	0.72	0.72
chr19:44414952	Wnt8b	Scd1	0.94	0.93	0.93
chr19:44414953	Wnt8b	Scd1	0.96	0.95	0.94
chr5:149008580	Hmgb1	Gm15409	0.63	0.63	0.58
chr9:21632184	Ldlr	Smarca4	0.75	0.70	0.68

## Discussion

In this study we asked a fundamental question regarding the epigenetic responses to different diets: what happens to DNA methylation changes in liver in response to a HFHS diet challenge and are these changes reversible when mice are fed a low-fat, weight loss diet? By identifying epigenetic changes and profiling weight loss phenotypes in a panel of genetically diverse mouse strains, we were able to evaluate this question in the context of variable gene-by-environment responses. Mouse studies of obesity often overlook genetic and phenotypic variability when discovering epigenetic mechanisms. By identifying diet-responsive CpGs across 45 strains, we were able to identify candidate methylation changes associated with a common response to overnutrition in liver. These changes could be used as a future metric for HFHS exposure agnostic of genetic background. The genes regulated by diet-responsive CpGs in this study are known to be involved in energy homeostasis. Once identified, we investigated the status of these diet-responsive CpGs in the livers of animals that were exposed to HFHS but returned to chow-diet and subsequently reduced their adiposity. We observed a strain-specific phenotype in weight loss, as only one strain of the five, A/J, did not return to chow-only levels of body fat after 4 weeks. And although most strains successfully lost enough body fat to be comparable to age matched, chow-only controls, a large fraction of diet-induced methylation changes remained significantly differentially methylated. This suggests that epigenetic changes revert more slowly than physiological changes, like adiposity.

One of the genes associated with the greatest number of diet-responsive CpGs and persistently differentially methylated after weight loss was *Cdk1*. The CpGs at the Chr10 region annotated only to *Cdk1* and no other nearby gene. Methylation at this locus was also significantly correlated with *Cdk1* expression. *Cdk1* is a gene for a kinase involved in cell entry into mitosis. *Cdk1* inhibition was recently discovered to enhance glucose sensing in pancreatic beta cells by regulating mitochondrial bioenergetics (30). Epigenetic regulation of *Cdk1* has not previously been implicated in a hepatic response to overnutrition and increased adiposity. Another prominent gene near diet-responsive CpGs was *Scd1*. *Scd1* is a well-studied gene involved in the biosynthesis of monounsaturated fatty acids, critical signaling molecules in energy homeostasis (3). A clinical study examining methylation at the promoter of *SCD1* in humans identified hypomethylation in obese individuals but an increase in methylation levels following Roux-en-Y Gastric Bypass surgery (31). The delayed reversal of obesity-induced methylation at *Scd1* in mice could be either due to the length of weight loss time observed in this study or an effect of rapid weight loss due to surgery in humans.

In this study, 4 weeks of chow diet reversal was sufficient to significantly restore body fat to previous levels in 4 of 5 strains studied. To date, there are few published weight loss studies in mice that use >2 genetically diverse strains to interrogate this phenotype. Leung et al. compared the hepatic chromatin states of C57BL/6J and A/J in the context of diet-induced obesity and weight loss for 16 weeks (23). The authors identified an obesity resistant and easily reverted phenotype in A/J compared to C57BL/6J animals fed the same obesogenic diet used in these studies. In alignment with this resistant phenotype, the authors identified less persistently accessible regions in A/J compared to C57BL/6J. We did not observe this pattern of epigenetic and body composition plasticity in the A/J strain over the course of our 10 week study, as the A/J strain behaved more similarly to the C57BL/6J cohort from Leung et al. Weight loss studies performed in C57BL/6J animals have also been inconclusive with regard to the reversibility of obesity induced changes, suggesting subtle differences in environment and experimental design may affect the reversion phenotype (24, 32).

In our study we observed that weight loss occurs more rapidly than DNA methylation changes. It is possible that some sites may eventually revert back to their chow state, but the time scale for this can be significantly longer than those for weight loss itself. With respect to the kinetics of epigenetic change, DNA methylation is considered to be the most stable epigenetic mark. Compared to chromatin remodeling mechanisms such as histone deacetylases and demethylases, *DNMT3A* the *de novo* DNA methylation enzyme, confers more persistent epigenetic changes by silencing gene expression with the slowest kinetics (33). This suggests that changes to DNA methylation during weight loss are less likely to be reversed within the same period of time as histone modifications or DNA accessibility. As a whole, most strains examined in this study efficiently reversed the obesity phenotype following reversion to a chow diet. However, methylation changes induced by the initial state of obesity are not quickly reversed at loci involved in energy homeostasis, even after adiposity is reversed.

The discordant dynamic between physiological and epigenetic plasticity observed in our study is reminiscent of persistent epigenetic aging acceleration in human obesity. The “epigenetic clock” model, developed by Horvath et al., associates the level of DNA methylation at dozens of CpGs with chronological age (10). This clock is often used to evaluate the effect of disease states and environmental exposures on tissue physiology by observing the changes in epigenetic age acceleration. Epigenetic age acceleration is a biomarker that is hypothesized to represent age-associated decline in tissue function (34). Obesity increases the acceleration of epigenetic aging in the liver, and this acceleration is not reversed following bariatric surgery (10). The accelerated clock markers in humans were enriched for CpGs in close proximity to genes involved in mitochondrial function and oxidative stress. These physiological processes are involved in insulin resistance, a prevalent phenotype in aged individuals that may play a role in the prolonged physiological reversal of obesity (35). Studies of diet induced obesity in mice identified persistent insulin resistance following short-term weight loss (22, 36, 37). Persistent epigenetic alterations have also previously been proposed as a “molecular memory” mechanism in gluconormalized diabetics with an increased risk for retinopathy and additional microvascular complications (38, 39). Taken together, these studies and our present work suggest that obesity-induced changes to DNA methylation at loci associated with energy homeostasis may be involved in unresolved metabolic adaptation, or epigenetic “memory” following weight loss. This adaptation has potential implications for predicting the success of weight loss, and subsequent risk of future weight regain.

## Data Availability Statement

The datasets generated for this study can be found in the Genome Expression Omnibus (GEO) GSE64770.

## Ethics Statement

The animal study was reviewed and approved by Institutional Care and Use Committee (IACUC) at University of California, Los Angeles.

## Author Contributions

BP, MM, and CE conceived the original ideas. BP and MM carried out the mouse experiments. CE performed sequencing and data analysis. AL and MP helped supervise the project. CE wrote the manuscript with support from AL and MP.

### Conflict of Interest

The authors declare that the research was conducted in the absence of any commercial or financial relationships that could be construed as a potential conflict of interest.

## Supplementary Material

Supplementary Figure 1 Examples of diet-induced methylation changes in all 45 HMDP strains at individual CpGs (*n* = 45). Each line represents the change in methylation by HFHS within a single strain. The CpGs on chromosome 15 and 19 are examples of a CpGs that trend toward hypomethylation after HFHS.

Supplementary Figure 2 **(A)** Shows the correlation of HFHS diet-induced changes between replicate experiments. Three strains, BALB/cJ, C57BL/6J, and DBA/2J were included in both analyses of HFHS diet-induced methylation changes. Data shown are 81 HFHS-responsive CpGs (as identified in the 45 strain study) shared across both studies in all 3 strains. X axis values represent the delta methylation by HFHS in the 45 strain cohort (exp. 1) and the Y axis values represent the delta methylation between strain averages in the 5 strain cohort (exp. 2). **(B)** Shows the correlation of *Scd1* fragment methylation with body fat across each diet group in weight loss cohort. Pearson correlation coefficients between log body fat percentage and methylation level at chromosome 19 locus identifies a decrease in the fraction of body-fat variation explained by methylation after weight loss.
