# Supplementary figures and images for: DNA Methylation Changes More Slowly Than Physiological States in Response to Weight Loss in Genetically Diverse Mouse Strains
Source: Front Endocrinol (Lausanne). 2019 Dec 20;10:882. doi: 10.3389/fendo.2019.00882 (PMC6933503; doi:10.3389/fendo.2019.00882)

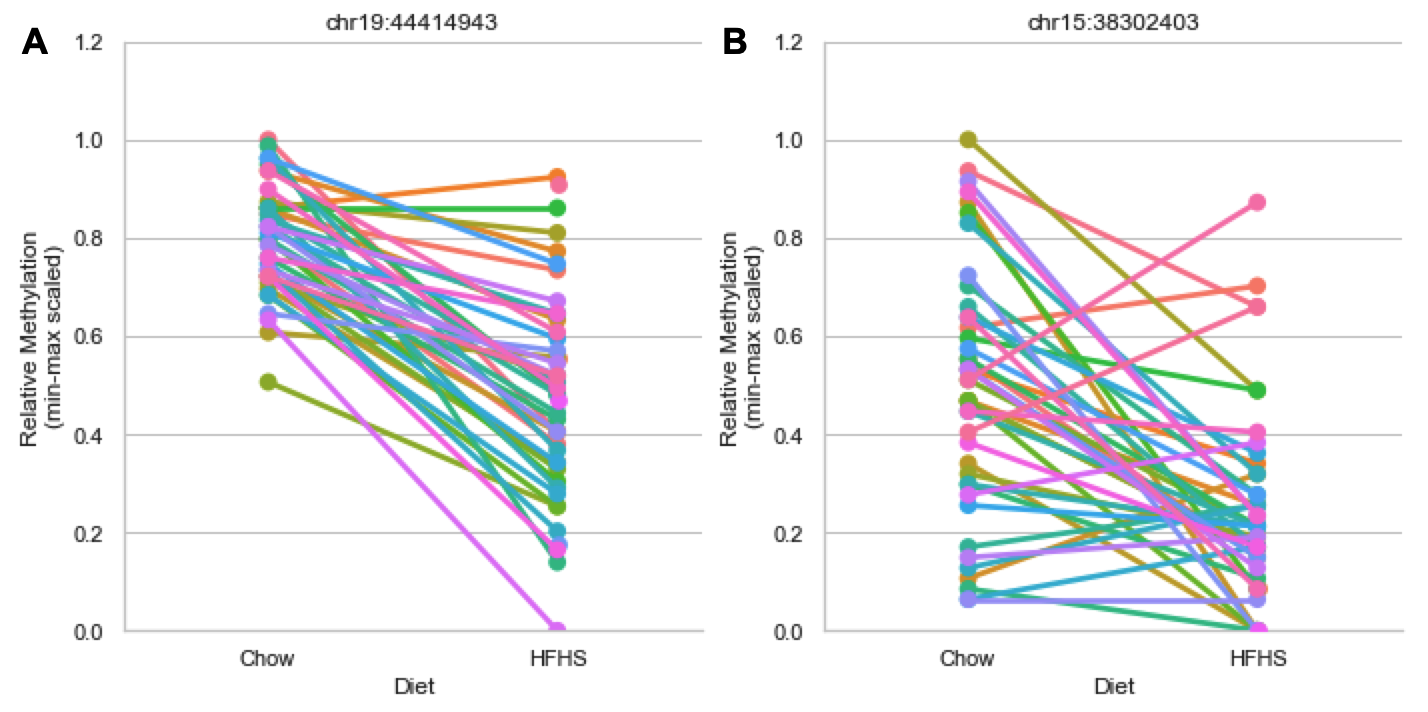

Supplement: Supplementary Figure 1 — Examples of diet-induced methylation changes in all 45 HMDP strains at individual CpGs (n = 45). Each line represents the change in methylation by HFHS within a single strain. The CpGs on chromosome 15 and 19 are examples of a CpGs that trend toward hypomethylation after HFHS. [file Image_1.png]

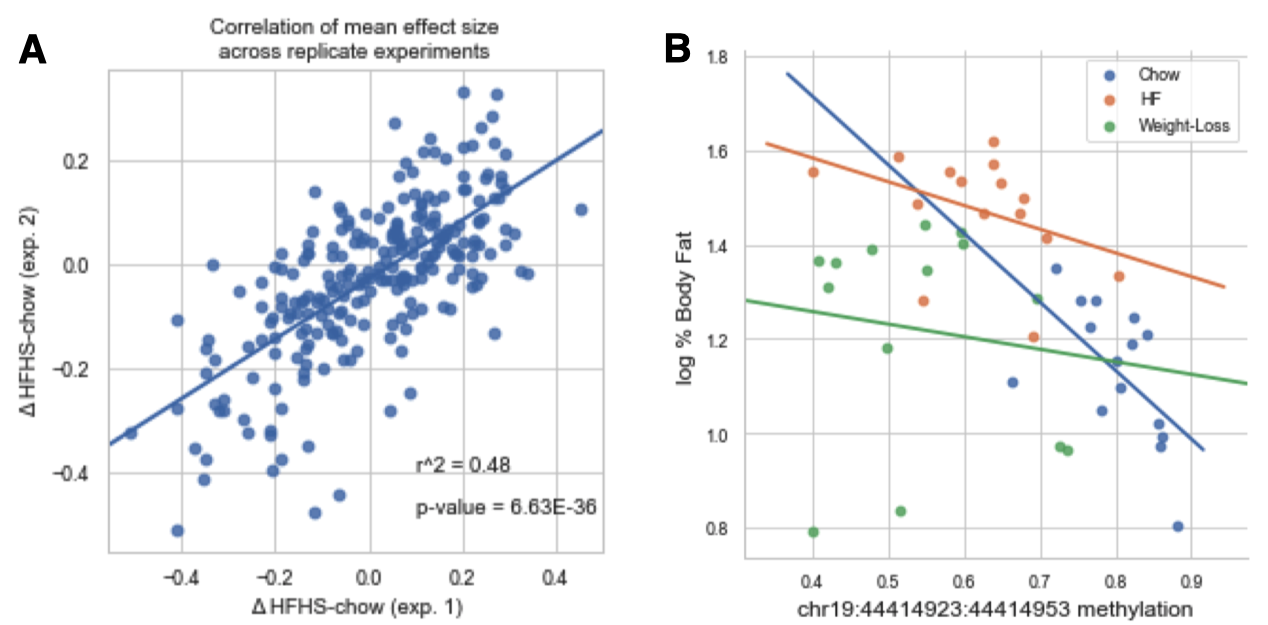

Supplement: Supplementary Figure 2 — (A) Shows the correlation of HFHS diet-induced changes between replicate experiments. Three strains, BALB/cJ, C57BL/6J, and DBA/2J were included in both analyses of HFHS diet-induced methylation changes. Data shown are 81 HFHS-responsive CpGs (as identified in the 45 strain study) shared across both studies in all 3 strains. X axis values represent the delta methylation by HFHS in the 45 strain cohort (exp. 1) and the Y axis values represent the delta methylation between strain averages in the 5 strain cohort (exp. 2). (B) Shows the correlation of Scd1 fragment methylation with body fat across each diet group in weight loss cohort. Pearson correlation coefficients between log body fat percentage and methylation level at chromosome 19 locus identifies a decrease in the fraction of body-fat variation explained by methylation after weight loss. [file Image_2.png]
